# Indoleamine-2,3-dioxygenase and Interleukin-6 associated with tumor response to neoadjuvant chemotherapy in breast cancer

**DOI:** 10.18632/oncotarget.22253

**Published:** 2017-11-01

**Authors:** Fangxuan Li, Lijuan Wei, Shixia Li, Juntian Liu

**Affiliations:** ^1^ Department of Cancer Prevention, Tianjin Medical University Cancer Institute and Hospital, National Clinical Research Center for Cancer, Tianjin's Clinical Research Center for Cancer, Key Laboratory of Cancer Prevention and Therapy, Tianjin 300060, China

**Keywords:** Indoleamine-2,3-dioxygenase, Interleukin-6, neoadjuvant chemotherapy, breast cancer, clinical response

## Abstract

**Purpose:**

Indoleamine-2,3-dioxygenase (IDO) and Interleukin-6 (IL-6) contribute to poor therapeutic effects, tumor relapse and aggressive tumor growth. IDO and IL-6 incorporate a positive feedback signal loop to maintain IDO and IL-6 constitutive expression and facilitate tumor progression.

**Results:**

IDO expression was associated with IL-6 expression and plasma IL-6 level (*P<*0.05). Concentrating on clinicopathological features prior neoadjuvant chemotherapy, both IDO expression and plasma IL-6 level were associated with clinical T stage and N stage (*P<*0.05). IL-6 expression was associated with clinical T stage (*P=*0.016). The co-expression of IDO/IL-6 was correlated with clinical T, N stage and estrogen receptor (ER) status (*P*<0.05). IDO, IL-6 expression, clinical T stage, pathological T stage, ER status and Luminal type were correlated with clinical response to neoadjuvant chemotherapy (*P*<0.05). Multivariate analysis showed that IDO expression were correlated with clinical response to neoadjuvant chemotherapy (*P=*0.034). IL-6 expression and pathological T stage were correlated with pCR (*P*<0.05). In the multivariate analysis, postoperative pathological T stage associated with pCR (*P=*0.041). In the prognostic analysis, only clinical T stage was significant correlated with overall survival (*P*=0.003).

**Materials and Methods:**

46 breast cancer patients received neoadjuvant chemotherapy enrolled in this study. Immunohistochemistry was applied for evaluating IDO and IL-6 expression in biopsy tissues prior neoadjuvant chemotherapy. Immunofluorescence was applied to observe the co-localization of IDO and IL-6. Serum IL-6 level was examined via ELISA. The associations between IDO, IL-6, Serum IL-6 level and clinicopathological features, response to neoadjuvant chemotherapy were analyzed.

**Conclusion:**

IDO and IL-6 expression associated with advanced breast cancer and poor response to neoadjuvant chemotherapy.

## INTRODUCTION

Worldwide, breast cancer is the most common malignant tumor in female [1]. Chemotherapy is predominantly used for breast cancer at stages II to stage IV, and is particularly beneficial for estrogen receptor negative (ER-) patients [2]. Neoadjuvant chemotherapy is a recent treatment regimen for breast cancer patients who were diagnosed with high risk non-metastatic breast cancer including locally advanced and inflammatory breast cancer. This approach was reported to shrink the tumor prior to mastectomy or lumpectomy [3], which can improve breast conserving rates, decrease the recurrence, prolong progression free survival (PFS) and overall survival (OS). What's more, in neoadjuvant chemotherapy, tumor size reduction and treatment tolerability could be evaluated, with potential for modifying chemotherapy regimens to increase rates of pathological complete response (pCR), which allowed the primary tumor response to serve as a mean of chemo-sensitivity estimation [4]. pCR is reported to be a useful prognostic marker, since patients who achieve pCR exhibit significant improvement in survival [5, 6].

The important role of the immune system in tumor occurrence and progression has been debated for many years. Swann et al had demonstrated malignant cells can escape the immunology surveillance via various ways. One of these important ways is immune suppression, which could depend on many tumor and host factors, involving different inflammatory molecules [7].

Interleukin-6 (IL-6) is one such inflammatory molecule. It is involved in the proliferation and differentiation of tumor cells and demonstrated to be high in serum and tumor tissues in series cancers, eg. colorectal cancer, breast cancer, prostate cancer [8]. It had confirmed that IL-6 contributes to poor therapeutic effect, tumor recurrence and aggressive tumor growth. In previous study, patients with higher serum IL-6 are generally associated with poorer prognosis, while lower level IL-6 is associated with better response to therapy [9]. It implies that malignant cells secret IL-6 as a protective mechanism against chemotherapy induced cell death [10, 11]. Series studies found that high level IL-6 cause resistance via mediating STAT3 activation [12] and inducing expression of the multidrug resistance gene and CYP450 enzymes through the janus kinase/signal transducers and activators of transcription (JAK/STAT) and phosphatidylinositol 3 kinase/protein kinase B (PI3K/AKT) pathways [13].

Indoleamine-2,3-dioxygenase (IDO) acts in tumor, stromal and immune cells to promote pathogenic inflammatory reaction which induce the immune escape of tumor cells [14, 15]. Muller et al. reported that IDO inhibition combined with chemotherapeutic drugs to effectively promote the suppressor of recurrent breast neoplasm [16]. In Okamoto et al's study, greater expression of IDO was confirmed not only in tumors from chemoresistant patients but also in chemoresistant ovarian cancer cell lines, suggesting that IDO may participate in chemosensitivity by intracellular mechanisms [17]. In glioblastoma's study, the IDO pathway contribute to complement dependent enhancement of chemo-radiation treatment for murine glioblastoma (GL261 tumors in syngeneic host mice) [18].

IDO was demonstrated to drive IL-6 production in lung cancer and metastatic breast cancer, while, downstream product of IDO metabolism, kynurenic acid, can potentiate IL-6 production by the aryl hydrocarbon receptor (AHR) [19]. Yet, IL-6 was reported, in turn to induce IDO expression through JAK/STAT signaling in rat hippocampus (Wistar rat, C57BL/6J wild type mice and B6.129/J^IDO-/-^ mice) [20]. Litzenburger et al. suggested that IDO-AHR-IL-6-STAT3 positive feedback signal loop maintain IDO and IL-6 expression in human cancer cells which may imply novel targets of this pathway promoting immunosuppression for cancer treatment [21].

Thus, in our study we evaluated the IDO and IL-6 expression of biopsy specimens and IL-6 level of serum collected prior to neoadjuvant chemotherapy based on clinical and pathological response to chemotherapy, to explore the association between IDO, IL-6 expression and clinicopathological features, response to neoadjuvant chemotherapy and prognosis of breast cancer patients.

## RESULTS

### The association between IDO, IL-6 expression and plasma IL-6 level

**Figure 1:**
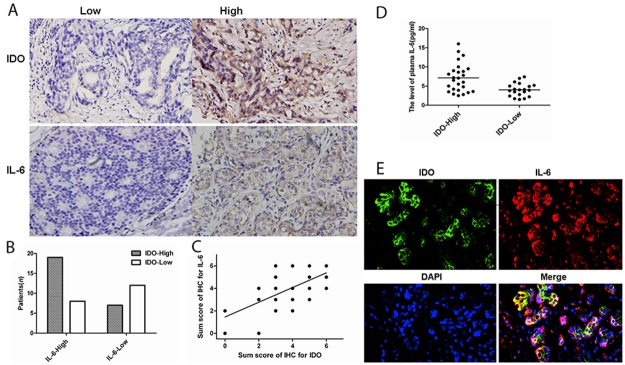
The association between IDO, IL-6 expression and plasma IL-6 level **(A)** The expression of IDO and IL-6 in tumor tissues. **(B)** Patients with high IDO expression more frequently had higher IL-6 high expression. **(C)** By Spearman's rank correlation test, it was found that the sum score of IDO correlated with the sum score of IL-6 (*r^2^*=0.665, *P*<0.001). **(D)** showed that the level of plasma IL-6 in patients with high IDO expression (7.183±3.678pg/mL, Range: 2.510~16.021pg/mL) was higher than patients with low IDO expression (4.030±1.713pg/mL, Range: 1.495~7.487pg/mL, *t*=3.487, *P=*0.001). **(E)** immunofluorescence double staining for co-localization of IDO and IL-6 in tumor tissues.

The Figure 1A showed the expression of IDO and IL-6 in tumor tissues. Figure 1B showed that the patients with high IDO expression more frequently had higher IL-6 high expression. From the total of 46 patients, 26 patients were high expression of IDO, 27 patients were high for IL-6 expression, and 19 patients were positive for both IDO and IL-6 expression. In Figure 1C, it was found that the sum score of IDO correlated with the sum score of IL-6 (*r^2^*=0.655, *P*<0.001) by Spearman's rank correlation test. Figure 1D showed that the level of plasma IL-6 in patients with high IDO expression (7.183±3.678pg/mL, Range: 2.510~16.021pg/mL) was higher than patients with low IDO expression (4.030±1.713pg/mL, Range: 1.495~7.487pg/mL, *t*=3.487, *P=*0.001). The IDO and IL-6 expression status in breast cancer tissues were assessed by immunofluorescence double staining as shown in Figure 1E. In the present study, the two-mixed primary antibody set were used and the images were visualized using two colors as green (IDO), red (IL-6). These color images were merged as yellow, which showed that IDO and IL-6 were co-localized in both IDO and IL-6 high expression tissues (*r^2^*=0.690, *P<*0.001).

### The association between IDO, IL-6 expression, plasma IL-6 level and clinicopathological features

We analyzed the association between IDO, IL-6 expression, plasma IL-6 level and clinicopathological features of breast cancer prior neoadjuvant chemotherapy. Both higher IDO expression and plasma IL-6 level were associated with T3+T4 stage and N1-N3 stage (*P<*0.05). Higher IL-6 expression in cancer tissues was more frequent in patients with T3+T4 stage (*P=*0.016), shown in Table 1.

**Table 1:** The association between IDO, IL-6 expression, plasma IL-6 level and clinicopathological features prior neoadjuvant chemotherapy

Clinicopathological features		IDO		*x^2^*	*P*	IL-6		*x^2^*	*P*	Plasma IL-6 *Mean*±SD	*t*	*P*
		Low	High			Low	High					
Age		45.931±12.701	50.211±7.812	1.429	0.106	48.000±10.951	48.520±9.702	0.169	0.866	7.113±4.668	0.213	0.186
Menopausal status	Pre-	9	13	0.113	0.774	9	13	0.003	1.000	7.592±3.927	0.852	0.398
	Post-	11	13			10	14			6.550±4.333		
History of gravidity	No	4	2	1.510	0.219	2	4	1.181	1.000	7.627±3.827	0.212	0.832
	Yes	16	24			17	23			7.195±4.733		
Family history of maligant	No	14	19	0.053	1.000	13	10	0.176	0.746	7.752±3.771	0.849	0.400
	Yes	6	7			6	7			7.634±4.612		
Clinical T stage	T1, T2	12	7	5.101	0.036	12	7	6.377	0.016	7.922±4.075	2.212	0.032
	T3, T4	8	19			7	20			5.004±4.842		
Clinical N stage	N0	8	3	5.033	0.038	6	5	1.046	0.484	4.804±4.765	2.102	0.042
	N1-N3	12	23			13	22			7.809±3.877		
Clinical stage	I, II	7	4	2.361	0.309	5	6	0.103	0.749	7.2927±4.271	0.362	0.718
	III, IV	13	22			14	21			6.775±4.0738		
Histological type	IDBC	17	21	0.141	1.000	16	22	0.058	1.000	7.112±4.018	0.150	0.881
	Other	3	5			3	5			6.875±4.2738		
ER-status	-	11	7	3.741	0.073	6	12	0.775	0.541	7.414±3.980	0.511	0.611
	+	9	19			13	15			6.9784±.1738		
PR	-	9	13	0.113	0.774	11	13	0.425	0.562	7.525±4.98	0.931	0.356
	+	11	13			8	14			6.1878±4.738		
Her-2	-	15	14	2.171	0.219	14	15	1.573	0.235	6.025±4.980	1.174	0.247
	+	5	12			5	12			7.781±4.738		
Ki67	-	9	9	0.512	0.550	9	9	0.992	0.373	5.871±3.738	1.460	0.150
	+	11	17			10	18			7.761±5.028		
P53 protein	-	9	12	0.006	1.000	9	12	0.038	1.000	5.973±4.527	1.600	0.246
	+	11	14			10	15			7.775±3.074		
Luminal type	Luminal	13	17	0.425	0.809	13	17	1.865	0.394	4.530±4.172	0.745	0.482
	Her-2	5	5			5	5			7.351±4.411		
	Basal-like	1	3			1	5			8.331±6.823		

IDBC: Invasive ductal breast cancer. For categorical variables, the *x^2^* value and *P* value were calculated by Chi-square tests. For continuous variables, values are given as mean ± standard deviation and compared with independent *t*-test.

Then, we analyzed the association between IDO/IL-6 co-expression and clinicopathological features prior neoadjuvant chemotherapy, which was both IDO and IL-6 high expression. The co-expression of IDO/IL-6 were correlated with advanced clinical T (*P*=0.022), N stage (*P*<0.001), clinical stage (*P*=0.002) and ER+ (*P*=0.039, shown in Table 2).

**Table 2:** The association between IDO/IL-6 co-expression and clinicopathological features prior neoadjuvant chemotherapy

Clinicopathological features		*n*	IDO-/IL-6-	IDO+/IL-6-	IDO-/IL-6+	IDO+/IL-6+	*x^2^*	*P*
Total (*n*)		46	13	6	7	20		
Age		46	48.38±12.21	47.17±8.542	41.29±11.91	51.05±7.619	1.716	0.178
Menopausal status	Pre-	22	7	2	2	11	2.146	0.543
	Post-	24	6	4	5	9		
History of gravidity	No	6	1	1	3	1	7.024	0.071
	Yes	40	12	5	4	19		
Family history of maligant	No	33	9	4	5	15	0.222	0.974
	Yes	13	4	2	2	5		
Clinical T stage	T1, T2	19	10	2	2	5	9.621	0.022
	T3, T4	27	3	4	5	15		
Clinical N stage	N0	11	2	4	5	0	21.519	<0.001
	N1-N3	35	11	2	2	20		
Clinical stage	I, II	11	2	3	5	1	15.382	0.002
	III, IV	35	11	3	2	19		
Histological type	IDBC	38	12	4	5	17	2.601	0.457
	Other	8	1	2	2	3		
ER	-	18	5	1	6	6	8.351	0.039
	+	28	8	5	1	14		
PR	-	22	8	3	3	8	1.551	0.671
	+	24	5	3	4	12		
Her-2	-	29	9	5	6	9	5.613	0.132
	+	17	4	1	1	11		
Ki67	-	18	6	3	3	6	1.308	0.727
	+	28	7	3	4	14		
P53	-	21	5	4	4	8	1.969	0.579
	+	25	8	2	3	12		
Luminal type	Luminal	30	8	5	4	13	3.585	0.733
	Her-2	10	4	1	1	4		
	Basal-like	6	1	0	2	3		

IDBC: Invasive ductal breast cancer. For categorical variables, the *x^2^* value and *P* value were calculated by Chi-square tests. For continuous variables, values are given as mean ± standard deviation and compared with independent *t*-test.

### The association between IDO, IL-6 expression, plasma IL-6 level and response to neoadjuvant chemotherapy

**Figure 2:**
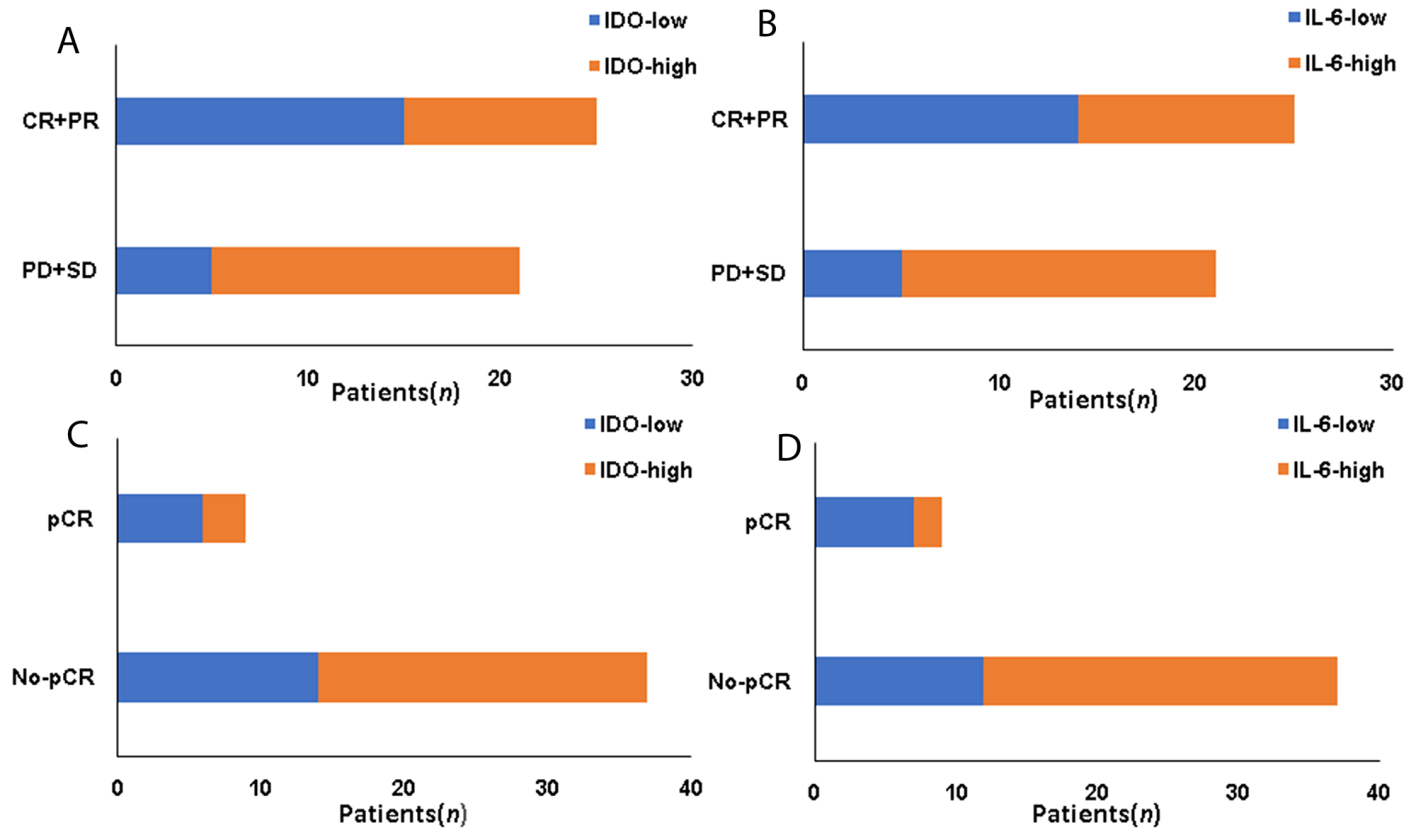
The association between IDO, IL-6 expression and response to neoadjuvant chemotherapy **(A)** The association between IDO and CR+PR. **(B)** The association between IL-6 and CR+PR. **(C)** The association between IDO and pCR. **(D)** The association between IL-6 and pCR.

In Table 3 and Figure 2, univariate analysis showed that IDO (*P*=0.017, Figure 2A), IL-6 expression (*P*=0.031, Figure 2B), clinical T stage (*P*=0.031), ER-status (*P*=0.014) and Luminal type (*P*=0.046) prior neoadjuvant chemotherapy and postoperative pathological T stage (*P*=0.042) were correlated with poor clinical response to neoadjuvant chemotherapy (*P<*0.05). In this study, *P*<0.05 was considered to be statistically significant; however, the statistical significance of IL-6 (*P=*0.031), clinical T stage (*P=*0.031), pathological T stage (*P=*0.042), luminal type (*P=*0.046) were borderline. Therefore, we enrolled IDO and ER status in the multivariate analysis. Multivariate logistic regression showed that IDO [*OR*=4.254(1.042~17.362), *P=*0.034] were correlated with clinical response to neoadjuvant chemotherapy.

**Table 3:** The association between IDO, IL-6 expression plasma IL-6 level and clinicopathological features with CR+PR

Clinicopathological features		*n*	CR+PR (n)	PD+SD (n)	*x^2^*	*P*	*OR (95%CI)*	*P*	*OR (95%CI)*	*P*
Age			48.52±11.27	48.05±10.26	0.877	0.472	0.995(0.939-1.005)	0.873		
Menopausal status	Pre-	22	14	8	1.446	0.253	0.484(0.148-1.578)	0.229		
	Post-	24	11	13			1			
History of gravidity	No	6	4	2	0.422	0.673	0.553(0.091-3.368)	0.520		
	Yes	40	21	19			1			
Family history of maligant	No	33	17	16	0.378	0.539	1.506(0.407-5.578)	0.540		
	Yes	13	8	5			1			
IDO	Low	20	15	5	6.083	0.019	0.208(0.028-0.752)	0.017	4.254 (1.042~17.362)	0.034
	High	26	10	16			1		1	
IL-6	Low	19	14	5	4.878	0.038	0.246(0.068-0.881)	0.031		
	High	27	11	16			1			
Plasma IL-6 level		46	6.681±4.033	8.891±3.879	1.904	0.063	0.980(0.957-1.003)	0.093		
IDO/IL-6	IDO-low/IL-6-low	13	12	1	10.650	0.014	0.056(0.006-0.515)	0.068		
	IDO-highIL-6-low	6	2	4			1.333(0.196-9.083)	0.011		
	IDO-low/IL-6-high	7	3	4			0.889(0.155-5.084)	0.769		
	IDO-high/IL-6-high	20	8	12			1	0.895		
Clinical T stage	T1, T2	19	14	5	4.878	0.038	4.073(1.635-14.632)	0.031		
	T3, T4	27	11	16			1			
Clinical N stage	N0	11	4	7	1.885	0.298	0.381 (0.094-1.548)	0.177		
	N1-N3	35	21	14			1			
Clinical stage	I+II	11	5	6	0.461	0.730	0.625(0.160-2.441)	0.499		
	III+IV	35	20	15			1			
Pathological T stage	pT1, T2	36	23	13	4.076	0.043	0.141(0.026-0.717)	0.042		
	pT3, T4	10	2	8			1			
Pathological N stage	pN0	8	4	4	0.074	1.000	0.810(0.176-3.125)	0.786		
	pN1-N3	38	21	17			1			
Pathological stage	I+II	16	8	8	0.187	0.760	0.765(0.226-2.583)	0.666		
	III+IV	30	17	13			1			
Histological type	IDBC	38	21	17	0.074	1.000	1.253(0.268-5.648)	0.786		
	Other	8	4	4			1			
ER	-	18	14	4	6.543	0.016	5.409(1.409-20.768)	0.014	3.739 (0.966~14.465)	0.056
	+	28	11	17			1			
PR	-	22	15	7	3.253	0.085	3.000(0.895-10.085)	0.075		
	+	24	10	14			1			
Her-2	-	29	14	15	1.166	0.363	0.509(0.148-1.747)	0.283		
	+	17	11	6			1			
Ki67	-	18	11	7	0.545	0.551	1.571(0.472-5.232)	0.461		
	+	28	14	14			1			
P53	-	21	11	10	0.060	1.000	0.846(0.270-2.771)	0.806		
	+	25	14	11			1			
Luminal type	Luminal	30	12	18	7.979	0.019	3.000(0.473-19.039)	0.046		
	Her-2	10	9	1			0.222(0.015-3.221)	0.244		
	Basal-like	6	4	2			1	0.270		
Chemotherapy regime	TAC/TEC	33	20	13	1.970	0.373	0.650(0.037-11.332)	0.386		
	TA/TE	11	4	7			1.750(0.084-36.287)	0.768		
	Other	2	1	1			1	0.718		
Chemotherapy cycles	2 cycles	33	21	12	4.060	0.056	0.254(0.164-1.004)	0.052		
	≥3 cycles	13	4	9			1			

IDBC: Invasive ductal breast cancer. For categorical variables, the *x^2^* value and *P* value were calculated by Chi-square tests. For continuous variables, values are given as mean ± standard deviation and compared with independent *t*-test.

Concentrated on the 9 patients who achieved pCR after neoadjuvant chemotherapy, only the higher IL-6 (*P*=0.028, Figure 2D) and postoperative pathological T stage (pT3+T4) associated with no-pCR (*P=*0.012). So, both of them were enrolled in the multivariate analysis. The multivariate analysis logistic regression showed that only pathological T stage correlated with no-pCR [*OR*=7.255(1.063-49.521), *P=*0.041, Table 4 and Figure 2].

**Table 4:** The association between IDO, IL-6 expression plasma IL-6 level and clinicopathological features with pCR

Clinicopathological features		*n*	pCR (n)	No-pCR (n)	*x^2^*	*P*	*OR (95%CI)*	*P*	*OR (95%CI)*	*P*
Age		46	48.52±11.27	48.05±10.26	0.877	0.472	1.031(1.955-1.114)	0.433		
Menopausal status	Pre-	22	4	18	0.051	0.821	1.184(0.274-5.121)	0.821		
	Post-	24	5	19			1			
History of gravidity	No	6	8	32	0.037	0.848	0.800(0.082-7.841)	0.848		
	Yes	40	1	5			1			
Family history of maligant	No	33	1	12	1.623	0.203	3.840(0.340-34.306)	0.229		
	Yes	13	8	25			1			
IDO	Low	20	6	14	2.448	0.149	3.316(0.607-18.124)	0.167		
	High	26	3	23			1			
IL-6	Low	19	7	12	6.140	0.022	7.579(1.060-66.813)	0.028	1.754(0.285-10.801)	0.545
	High	27	2	25			1		1	
Plasma IL-6 level		46	5.775±2.0738	7.2927±3.527	1.232	0.224	1.006(0.982-1.031)	0.616		
IDO/IL-6	IDO-low/IL-6-low	13	12	1	10.650	0.014	0.155(0.017-1.450)	0.359		
	IDO-highIL-6-low	6	2	4			0.000	0.102		
	IDO-low/IL-6-high	7	3	4			0.310(0.031-3.111)	0.999		
	IDO-high/IL-6-high	20	8	12			1	0.319		
Clinical T stage	T1, T2	19	4	15	0.461	0.831	0.852(0.196-3.705)	0.831		
	T3, T4	27	5	22			1			
Clinical N stage	N0	11	1	10	1.008	0.421	2.963 (0.328-26.790)	0.334		
	N1-N3	35	8	27			1			
Clinical stage	I+II	11	1	10	1.008	0.235	2.963 (0.328-26.790)	0.334		
	III+IV	35	8	27			1			
Pathological T stage	pT0, T1, T2	36	4	32	7.521	0.006	8.000(1.587-40.332)	0.012	7.255(1.063-49.521)	0.041
	pT3, T4	10	5	5			1		1	
Pathological N stage	pN0	8	2	6	0.182	0.670	0.677(0.112-4.091)	0.671		
	pN1-N3	38	7	31						
Pathological stage	I+II	16	8	8	0.187	0.760	0.338(0.076-1.505)	0.155		
	III+IV	30	17	13			1			
Histological type	IDBC	38	5	11	2.128	0.124	0.000	0.999		
	Other	8	4	24			1			
ER	-	18	2	16	3.883	0.055	6.880(0.771-59.980)	0.074		
	+	28	7	21			1			
PR	-	22	4	18	0.051	0.821	1.184(0.274-5.121)	0.821		
	+	24	5	19			1			
Her-2	-	29	5	24	0.269	0.604	1.477(0.337-6.474)	0.605		
	+	17	4	13			1			
Ki67	-	18	4	14	0.133	0.716	0.761(0.174-3.310)	0.716		
	+	28	5	23			1			
P53	-	21	2	19	2.476	0.116	3.694(0.676-20.694)	0.132		
	+	25	7	18			1			
Luminal type	Luminal	30	6	24	1.308	0.520	0.500(0.073-3.406)	0.541		
	Her-2	10	1	9			0.222(0.015-3.221)	0.479		
	Basal-like	6	2	4			1	0.270		
Chemotherapy regime	TAC/TEC	33	8	25	1.712	0.425	5.169×10^9^	0.586		
	TA/TE	11	1	10			1.615×10^9^	0.999		
	Other	2	0	2			1	0.999		
Chemotherapy cycles	2 cycles	33	8	25	1.623	0.410	0.260(0.029-2.327)	0.229		
	≥3 cycles	13	1	2			1			

IDBC: Invasive ductal breast cancer. For categorical variables, the *x^2^* value and *P* value were calculated by Chi-square tests. For continuous variables, values are given as mean ± standard deviation and compared with independent *t-*test.

### Prognostic analysis

**Figure 3:**
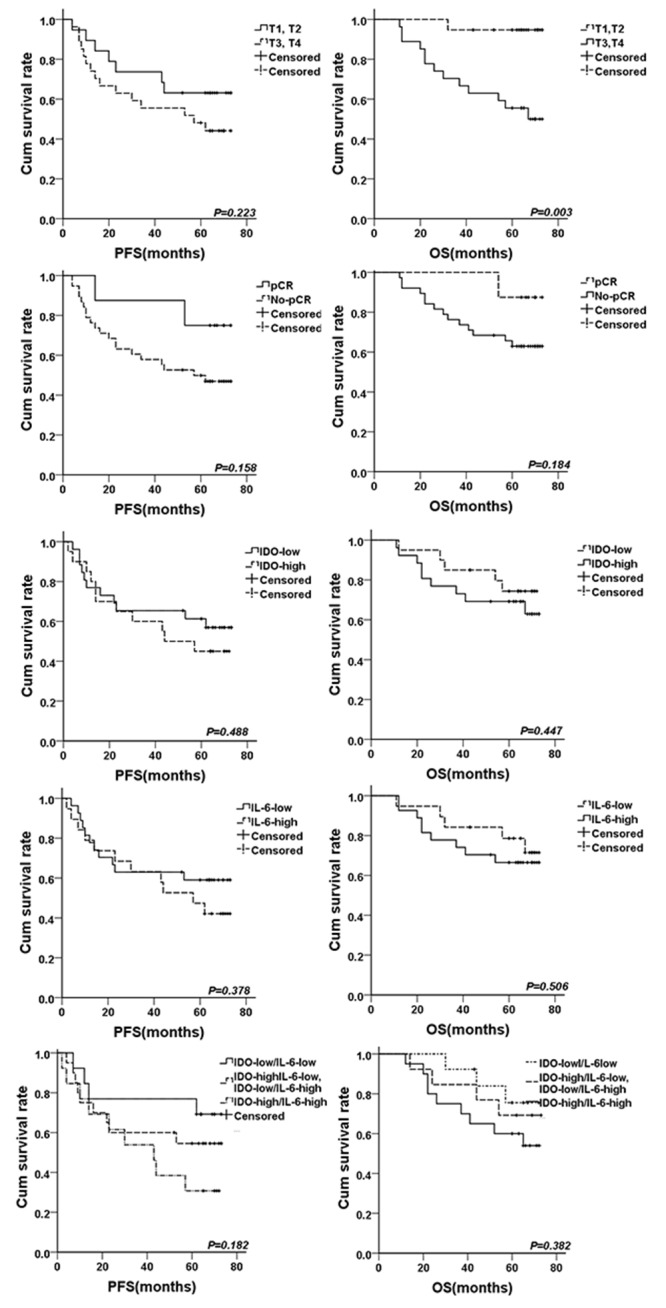
The survival curves for breast cancer patients treated by neoadjuvant chemotherapy The survival curves for PFS and OS according to different clinical T stage, pCR, IDO expression, IL-6 expression, IDO/IL-6 co-expression were shown.

In the survival analysis, only clinical T stage was significant correlated with overall survival time (OS, *P=*0.003). Concentrating on IDO and IL-6, the patients with high IDO or IL-6 expression had poorer prognosis in comparing with low IDO (*P=*0.447 for OS and *P*=0.488 for PFS) and IL-6 expression (*P*=0.506 for OS and *P*=0.378 for PFS), patients with co-expressions of IDO/IL-6 had shortest survival time when compared with low expression of IDO, IL-6 alone or both (*P=*0.382 for OS and *P*=0.182 for PFS). And, patients who obtained pCR had better survival than no-pCR patients (*P=*0.184 for OS and *P*=0.158 for PFS). However, all these survival differences were not statistically significant, shown in Figure 3.

## DISCUSSION

Constitutive IDO activity in tumors is a core mechanism regulating immune toleration and thus deemed as an attractive therapeutic target to recover immunity against cancer. In this study, the IDO expression was correlated with advanced clinical T and N stage prior neoadjuvant chemotherapy, which was consistent with previous studies [27]. In Soliman et al's study, IDO expression was higher in ER+ tumors compared to ER-tumors [22].

Okamoto et al reported that greater expression of IDO was demonstrated both in chemoresistant ovarian cancer patients and chemoresistant ovarian cancer cell lines, suggesting that IDO may participate in chemosensitivity via intracellular pathways [17]. IDO expression was down-regulated by nitric oxide, which is known to mediate chemosensitivity in tumor cells by salvaging product of mass cytosolic superoxide anions [23]. Therefore, Okamoto et al. suggested that the expression of IDO may be an accompanied phenomenon to other promoting mechanisms of chemoresistance, for example nitric oxide production, but not induce chemoresistance directly [17]. Muller et al. reported that IDO inhibitor combined with different chemotherapeutic drugs can induce regression of recurrent breast cancers more effectively [16]. In addition, Inaba et al confirmed that treatment with 1-MT, the inhibitor of IDO1, combined with paclitaxel congenially prolonged mouse survival compared to treatment with paclitaxel alone in an IDO1 high expressing ovarian cancer peritoneal carcinomatosis female C57BL/6 mice [24]. Salvador et al reported that combination 1-MT with paclitaxel would promote remodeling of T lymphocyte proliferation capability and its cytotoxic response [25].

All these reports indicated that IDO is positively associated with chemotherapy resistance and impaired survival. Muller et al. indicated that Bin1 loss increased the STAT1 and NF-kB dependent IDO expression [16], while the NF-kB activation suppresses the apoptotic potential of chemotherapeutic agents [26]. In our study, high IDO expression correlated with poorer clinical response (PD+SD) to neoadjuvant chemotherapy.

Higher levels of serum IL-6 are associated with aggressive cancer and response to therapies in series malignant tumors. Patients with high levels of serum IL-6 are also generally associated with poor prognosis and shorter survival [8, 27, 36, 41]. In this study, higher plasma IL-6 level was associated with advanced clinical T stage and N stage. IL-6 expression of cancer tissues was associated with advanced clinical T stage.

IL-6 secreted from either cancer cells or tumor microenvironment (immune cells and tumor stromal cells) not only facilitates tumor growth but also acts as a major barrier in achieving therapeutic efficacy [28]. Serum IL-6 levels have been reported to be associated with a poor prognosis and treatment failure in patient with many different carcinomas [29]. In prostate cancer, inhibition of IL-6 secretion increases the sensitivity of prostate cancer cells to anticancer drugs [30]. Previous study found that prostate cancer patients who responded to docetaxel and zolendronic acid therapy had a 35% decrease in overall serum IL-6 levels, while patients who did not respond had an increasing in serum IL-6 levels, which implied IL-6 regulate chemo-resistance in prostate cancer [8, 11]. The autocrine secretion of IL-6 by tumor cells is also confirmed to contribute to chemo-resistance; further studies demonstrated that drug sensitive tumor cells do not or low express IL-6, yet, multidrug resistant breast cancer cells secreted more IL-6 [31]. In this study, the low IL-6 expression was also associated with both clinical response and pathological response (PR+CR and pCR).

IL-6-mediated STAT3 activation has been reported to induce chemotherapy resistance in tumors by several pathways [11, 12]. IL-6 induced drug resistance is associated with increased expression of the multidrug resistance gene, mdr1, and upregulation of C/EBPβ and C/EBPδ (CCAAT enhancer-binding protein family of transcription factors) [31]. In Patel et al. study, IL-6 secreted from stromal cells induces CYP2E1 and CYP1B1 expression through the JAK/STAT and PI3K/AKT pathways, which causes tumor occurrence and progression by chemical carcinogens [13]. IL-6 also down regulate clinical outcome by maintaining abundant therapeutic resistant cancer stem cells which is play crucial role in tumor reoccurrence and resistance [32].

IDO was demonstrated to drive IL-6 production in lung cancer and metastatic breast cancer (Lox-Stop-Lox Kras^G12D^ transgenic mice) [33], while IL-6 was confirmed to promote IDO expression via JAK/STAT signaling pathway [20]. In this study, high IDO expression was correlated with high IL-6 expression both in breast cancer tissues and serum. Immunofluorescence double staining showed that IDO and IL-6 can be co-localized in IDO and IL-6 high expression tissues. Litzenburger et al suggested that the IDO-AHR-IL-6-STAT3 transcriptional loop represents a complex network of positive feedback [21]. In immune research, Hofer et al found that this positive feedback loop can cooperate a bistable system [34], which can be in a steady off-state, and all composes of this loop have only basal activity under the threshold for self-amplification, or in a steady on-state, the high activity of all composes is self maintained by positive feedback and limited only by sustaining degradation and inactivation [21]. We analyzed the association between IDO/IL-6 co-expression and clinicopathological features prior neoadjuvant chemotherapy. The co-expression of IDO/IL-6 was correlated with advanced clinical T, N stage and ER+.

In breast cancer tissues, IDO expression was correlated with IL-6 expression. Both of them were associated with clinicopathological features and response to chemotherapy. These findings suggested that IDO and IL-6 play important role in cancer progress and chemotherapy efficacy which maybe predictable markers for the chemotherapy response to aid clinical decision-making of more effective therapies for breast cancer. Additionally, immunotherapy therapies which inhibit IL-6 and IDO maybe rational agents of comprehensive treatment to improve outcome of chemotherapy. Particularly, agents targeting IDO (epacadostat, indoximod et al) as a standalone therapeutic agent often fails to prevent disease progression. However, IDO inhibitors have been evaluated for their ability to improve the efficacy of multiple chemotherapeutics, and some combinatorial regimens had promising results in preclinical studies [35].

But this study had many limitations. Firstly, this was a retrospective study, which can't randomly select patients and set control groups. What's more, research sample is relatively less, especially, only 9 patients achieved pCR, and all participants were from our center, which contributed to few significant difference of pCR analysis and prognostic analysis. In order to a more practical result, further clinical researches with larger sample and from multi-center are required. In addition, these data are only correlative analysis and further experimental study of molecular biology is required to reveal the interaction mechanisms of IDO and IL-6.

## MATERIALS AND METHODS

### Patients

This study was a retrospective study followed the REMARK criteria for biomarkers [35]. Inclusive criteria of patients included: (1) Patients with breast cancer registered in the Department of Breast Oncology of Tianjin Medical University Cancer Institute and Hospital between 1^st^, January, 2011-31^st^, August, 2011, and completed at least 5 years following-up; (2) Patients underwent coarse needle biopsies prior neoadjuvant chemotherapy and obtained at least three tissue cylinders for repeating at least triple H&E staining in pathological examination and immunohistochemical staining; (3) All the patients were histological-proven adenocarcinoma and had complete information on immunohistochemistry, including estrogen receptor (ER), progesterone receptor (PR), Her-2, P53, and Ki67; (4) All patients were administrated different cycles of planned-dose neoadjuvant according to a multi-agent chemotherapy protocols including TAC (docetaxel/ doxorubicin/ cyclophosphamide), TEC (docetaxel/ epirubicin/ cyclophosphamide), TA (docetaxel/ pirarubicin), TE (docetaxel/ epirubicin), EC (epirubicin/ cyclophosphamide), CEF (cyclophosphamide/ epirubicin/ fluorouracil). (5) All patients were implemented with R0 resection of mastectomy or lumpectomy with either ipsilateral sentinel lymph node biopsy or axillary dissection after evaluating the curative effect of neoadjuvant chemotherapy; (6). Surgical pathology reports were reviewed according to the 7^th^ edition of the AJCC Cancer Staging Manual. Thus, there were 46 patients enrolled in this study. The summary of clinicopathological characteristics were shown in Table 5.

**Table 5:** The summary of clinicopathological characteristics

Clinicopathological characteristics		*n*
Age (*n*, Mean±SD year)		46(48.33±13.16)
Menopausal status	Pre-/post	22/ 24
History of gravidity	No/ Yes	6/40
Family history of maligant	No/ Yes	33/13
Clinical T stage	T1/ T2/ T3/ T4	1/18/14/13
Clinical N stage	N0/ N1/ N2/ N3	11/10/12/13
Clinical stage	I/II/ III/IV	1/10/35/0
Pathological T stage	pT0/ T1/ T2/ T3/ T4	2/20/14/8/2
Pathological N stage	pN0/ N1/ N2/ N3	8/18/11/9
Pathological stage	I/II/III/IV	5/22/19/0
Histological type	IDBC/ ILC/ IPC/IBC	38/5/1/1
ER	-/+	18/28
PR	-/+	22/24
Her-2	-/+	29/17
Ki67	-/+	18/28
P53	-/+	21/25
Luminal subtypes	Luminal/Her-2/Basal-like	30/10/6
Chemotherapy regime	TAC or TEC/ TA or TE/EC/CMF	33/11/1/1
Chemotherapy cycles	2 cycles/≥3 cycles	33/13

IDBC: Invasive ductal breast cancer; ILC: Invasive lobular carcinoma; IPC: Invasive papillary carcinoma; IBC: Inflammatory breast cancer. Luminal subtypes was by immunohistochemical method and included HER2 negative luminal type (luminal: HER2-negative and hormone receptor positive), HER2 positive type (HER2: HER2 positive irrespective of hormone receptors status), and triple negative (TN) type (Basal-like: negative for hormone receptors and HER2).

The Ethics Committee of Tianjin Cancer Institute and Hospital approved this research project. Written consents were obtained from each patient.

### Immunohistochemistry (IHC) for IDO and IL-6 expression

Formaldehyde-fixed, paraffin-embedded tissue samples obtained by coarse needle biopsy and mastectomy were sectioned into 4μm slices and affixed on glass slides. The immunohistochemical staining was performed according to the instruction manuals. After being heated for half an hour at 56°C, the slides were deparaffinized in xylene and rehydrated through graded alcohol. Antigens were retrieved by heating in citrate buffer for 3 minutes and endogenous peroxidase activity was quenched in a bath of methanol and hydrogen peroxide for 30 minutes. The slides were incubated with mouse anti-human IDO monoclonal antibody (Chemicon Corporation, MA, USA) at concentration of 1:300 or mouse anti-human IL-6 monoclonal antibody (Biolegend company, San Diego, CA) at concentration of 1:200 at 4°C overnight. The antibody were detected by a biotinylated secondary antibody (goat anti-mouse IgG-HRP, sc-2302, Santa Cruz, Cali, USA) labeled with streptavidin-horseradish peroxidase (HRP), a DAB staining kit was used for the visualization of immune reactive cells. IDO antibody IHC of human placenta were positive control, Mouse/rabbit isotype IgG1 were used as the negative controls. Positive cells were stained brownish yellow in the cytoplasm. For semi-quantitative analysis, staining rate (SR) and staining index (SI) both were indicators to describe protein expression of IDO and IL-6. The SRs referred to the percentages of positive samples in all samples. IDO and IL-6 expression score was separated from 0 to 3 (0=5% of tumor cells were stained; 1=5-30% were stained; 2=30-70% were stained; 3=70% were stained). The SIs was determined upon the average of at least five high-powered fields (400×magnification) and separated from 0 to 3 (0= no staining; 1=mild staining; 2=moderate staining; 3=strong staining). The last score was the sum of two parts above. The low expression was defined the last score≤3, The high expression was defined the last score>3 [37]. All samples were reviewed by experienced pathologists who were blinded to the identity of the specimens. The detailed SR and SI for each case were shown in Supplementary Table 1.

### Double-labeling immunofluorescence method

To examine the co-localization of IDO and IL-6, a double immunofluorescence study was performed as described previously [22]. The tissue section displayed strong positive expression in immunohistochemistry analysis was selected for the double-labeling immunofluorescence method. Paraffin sections were deparaffinized, microwaved and then incubated with the primary antibodies [mouse anti-human IDO monoclonal antibody (Chemicon Corporation, MA, USA) at concentration of 1:300 or mouse anti-human IL-6 monoclonal antibody (Biolegend company, San Diego, CA) at concentration of 1:200] overnight at room temperature. Then, the sections were incubated with fluorescent secondary antibodies [Alexa 594-labeled goat antibody against mouse IgG and Alexa 488-labeled goat antibody against rabbit IgG (1:400 each, Molecular Probes Inc., Eugene, OR, USA)] for 3 hours at room temperature. The stained sections were examined under a fluorescent microscope (BX53, Olympus, Tokyo, Japan).

### ELISA for serum IL-6 level

Blood was sampled from patients the day before coarse needle biopsy. After 30 min of coagulation, samples were centrifuged in a refrigerated centrifuge at 4°C for 10 min at 2000 rpm. Samples were stored in aliquots of 250 μL at −80°C until use. We analyzed serum IL-6 using ELISA kits (Genzyme, Cambridge, MA) following the manufacturer's instructions. The optical density (OD) was measured at 450nm (630nm as reference) by means of an Organon Teknika Microwell system (Reader 230s, Germany). A standard curve was obtained based on serial dilutions of r-IL-6, ranging from 1ng/mL to 250ng/mL. The results were expressed as concentrations of IL-6 (ng/mL) extrapolated from the standard curve. In view of determining the detection specificity, three positive serum samples were submitted to a neutralization test.

### Pathologic evaluation

The initial core biopsy sample of the primary tumor was evaluated using standard hematoxylin and eosin staining, IHC, and fluorescence or chromogenic in situ hybridization (FISH) (or both) to determine the histological subtype, the Ki67 index, and the status of ER, PR, and Her-2. Cut-off values for ER, PR, and Ki67 were 10%, 10%, and 20%, respectively [38]. Her-2 was scored for the intensity and the completeness of cell membrane staining (-, no staining; +, weak partial membranous staining in more than 10 % tumor cells;++, moderately complete membrane staining in more than or equal to 10 % tumor cells or strong complete membranous staining in less than or equal to 10 % of tumor cells; +++, strong complete membranous staining in more than 10 % of tumor cells). Her-2 (+++) was defined as positive. FISH assay was performed in cases with ++ immunoreactivity [39]. The luminal subtypes were based on IHC of ER, PR and Her-2 [40].

### Response to neoadjuvant chemotherapy

Clinical response was assessed based on a physical examination, mammography, and ultrasonography according to the Response Evaluation Criteria in Solid Tumors (RECIST 1.1). A clinical complete response (CR) was defined as the disappearance of all known lesions; a clinically partial response (PR) was defined as a ≥30% reduction in the sum of the longest diameter of the primary lesion; progressive disease (PD) was defined as a ≥20% increase in the sum of the longest diameter of the primary lesion; and stable disease (SD) was defined as neither sufficient shrinkage to qualify for cPR nor sufficient increase to qualify for PD [40].

pCR was defined as no evidence of invasive carcinoma in the breast at the time of surgery in line with the criteria of the National Surgical Adjuvant Breast and Bowel Project B-18 [42].

### Statistical analyses

The Chi-square and the Fisher's exact tests were used for categorical variables. Continuous variables presented as difference between groups were assessed by independent *t* test. The spearman’ rank-order test and liner regression analysis was used to assess correlations between continuous variables, while Pearson's test was used to assess correlations between continuous variables and classification of variables. The Logistic regression was applied in analysis the correlation between clinical features and response of neoadjuvant chemotherapy. The factors that had significant difference in univariate analysis were included in the multivariate logistic regression analysis. The survival curves were analyzed via Kaplan-Meier and Log-rank test. A *P* value<0.05 was considered statistically significance. The statistical analysis was performed using SPSS 17.0 software package.

## SUPPLEMENTARY MATERIALS FIGURES AND TABLES




