# Supplementary material for: Indoleamine-2,3-dioxygenase and Interleukin-6 associated with tumor response to neoadjuvant chemotherapy in breast cancer
Source: Oncotarget. 2017 Nov 1;8(64):107844–58. doi: 10.18632/oncotarget.22253 (PMC5746108; doi:10.18632/oncotarget.22253)
Supplement: Supplementary file 1 [file oncotarget-08-107844-s001.pdf]

Indoleamine-2,3-dioxygenase and Interleukin-6 associated with tumor response to neoadjuvant chemotherapy in breast cancer

SUPPLEMENTARY MATERIALS

Supplementary Table 1: The detailed serum IL-6 level, SR and SI in IHC for IDO and IL-6

See Supplementary File 1
